# Application of the Intraoral Scanner in the Diagnosis of Dental Wear: An In Vivo Study of Tooth Wear Analysis

**DOI:** 10.3390/ijerph19084481

**Published:** 2022-04-08

**Authors:** Victor Díaz-Flores García, Yolanda Freire, Susana David Fernández, Beatriz Tomás Murillo, Margarita Gómez Sánchez

**Affiliations:** 1 Department of Pre-Clinic Dentistry, School of Biomedical Sciences, Universidad Europea de Madrid, Calle Tajo s/n, Villaviciosa de Odón, 28670 Madrid, Spain; yolanda.freire@universidadeuropea.es (Y.F.); beatriz.tomas@universidadeuropea.es (B.T.M.); margarita.gomez2@universidadeuropea.es (M.G.S.); 2 Department of Clinical Dentistry, School of Biomedical Sciences, Universidad Europea de Madrid, Calle Tajo s/n, Villaviciosa de Odón, 28670 Madrid, Spain; susana.david@universidadeuropea.es

**Keywords:** tooth wear, intraoral scanner, digital dentistry

## Abstract

In recent years, there has been an increase in the incidence of dental wear; thus, an early diagnosis is important. Conventional methods of diagnosis are based primarily on the visual abilities of the dentist, and therefore the use of new technologies for the detection of dental wear may be very useful. The aim of the study was to analyze the sensitivity and specificity of the intraoral scanner for measuring dental wear, as well as to evaluate patients’ satisfaction with the use of the scanner. The study was conducted with 46 volunteers who underwent three intraoral analyses: a first baseline scanning, a second scanning after 6 months and a final scanning after one year performed by four operators divided into two groups. One of the operators performed the visual analysis of dental wear, and the other performed the analysis using the intraoral scanner 3M™ True Definition intraoral scanner (ESPE, Seefeld, Germany). The data obtained from the intraoral scanner showed levels of specificity and sensitivity that enable the intraoral scanner to be used as a diagnostic tool in the assessment of tooth wear. The participants also showed a high degree of satisfaction with the scanner as a communication tool.

## 1. Introduction

Tooth wear is a very broad term that was defined by Eccles [1] in 1982 as “pathological loss of tooth tissue by a disease progress other than dental caries”. Tooth erosion involves the loss of hard tissue caused by chemical dissolution without the intervention of oral bacteria. Dental erosion can be classified into two main groups: intrinsic, caused by stomach acids, and extrinsic, when the acids come from elements ingested in the diet [2]. Physiological tooth wear is a slow and progressive process. It starts at the cusp tips and incisal edges, leading to the disappearance of mamelons [3]. However, considering that the increase in quality of life and tooth survival is greater than it was decades ago [4], edentulism is becoming less and less frequent [5]. This situation contributes to the increased aging of dental tissues [4], which implies that tooth wear is a problem of great importance.

Early diagnosis of tooth wear is important both because of its high incidence and because of its adverse consequences [6]. To reach the correct diagnosis of these lesions, it is important to know the clinical features of each type of wear. Following Grippo’s proposal [7], hard tissue lesions can be classified into four categories: attrition (loss by interarctational mechanical action), abrasion (by non-occlusal mechanical reason), abfraction (occlusal overloads), and erosion (by endogenous or exogenous nonbacterial chemical factors) [8].

Many indices have been proposed to evaluate, grade, and diagnose tooth wear and lost tissue; however, none of them are accepted by the scientific community as a “gold standard” [9]. The most widely used method is the one proposed by Smith and Knight, which bases its diagnosis on the visual evaluation—directly in the mouth of the subject to be analyzed [10]. However, there are also other indirect methods, such as photographic or plaster models. These methods have limitations such as standardization or technique or observer error—a key circumstance when micron differences between samples are to be observed—which is a problem for patient safety [11].

In the search for a more objective system for the evaluation of tooth wear, different methods have been proposed, such as profilometers, predictability models, 3D laser confocal microscopy, or virtual models, whose application in daily clinical practice is very costly and technically complex [12,13,14].

Considering this circumstance and limitations of the classic methods, intraoral scanner technology is presented as an alternative for the diagnosis of tooth wear. Intraoral scanners are becoming more common in dental practices [15] and are currently marketed to dental professionals; their size and price make their common presence in today’s dental practice possible [16]. CAD/CAM (Computer-Aided Design and Computer-Aided Manufacturing) technology was introduced in dentistry in the early 1970s and since then has undergone a major evolution [17]. CAD/CAM systems consist of an information acquisition unit, software, and a milling unit [18]. It is the data acquisition unit that allows the collection of intraoral data [19] and therefore allows an accurate recording of the dental anatomy and morphology. In addition, it provides a stable and real measurement, avoids possible human errors, and facilitates the objective handling of the data [20].

There are several intraoral CAD/CAM systems on the market, differing in terms of operation, light source, need for surface conditioning, or format of the resulting file [18]. Scanners, moreover, present advantages in terms of the application of ecological sustainability in the dental practice [21] and dental patient safety by being less invasive than traditional methods of dental registration [22]. Recently, the use of intraoral scanners has been proposed for the monitoring, detection, and quantification of tooth wear [23]. Therefore, the aim of the present study was to evaluate the sensitivity and specificity of the scanner for measuring dental wear, as well as to analyze the degree of patient satisfaction in the use of the dental scanner as a diagnosis communication tool.

## 2. Materials and Methods

### 2.1. Patient Selection

Sixty-five university students from the Faculty of Biomedical and Health Sciences (European University of Madrid, Spain) were selected to carry out the tooth wear registry. Forty-six volunteers (32 men and 14 women) met the inclusion and exclusion criteria established in the study (Table 1). The age of the participants ranged from 20 to 34 years. All subjects gave their informed consent for inclusion before they participated in the study. The study was conducted in accordance with the Declaration of Helsinki, and the protocol was approved by the Comité Ético de Investigación Clínica Regional de la Comunidad de Madrid (Project identification code Desgaste-UE).

### 2.2. Clinical Procedures

The registration of the arches of the study participants (*n* = 46) was carried out by four clinical experts, who were distributed in two groups of two persons each. In each team, one examiner was dedicated exclusively to the scans and the other to the visual examination of the patients and information from the satisfaction surveys, each item of information being collected separately and always in the same order. The information from the surveys was recorded first, followed by the visual examination in order not to be influenced by the scanning.

The investigators in charge of the visual part were trained in consensus previously in the use of the Smith and Knight index until the same diagnostic ability was achieved. The examiners in charge of the scans received a 30 h training course on the use and specifications of the scanner.

Before starting the data collection, the volunteers underwent a clinical examination and, if necessary, dental prophylaxis was provided to avoid interference with the bacterial plaque in the data with a brush and prophylactic paste mounted on a micromotor (KaVo Dental GmbH™, Biberach, Germany) and, if tartar was present, it was removed with ultrasound (Satelec Acteon™, Merignac, France) and a nº1 Universal tip (Satelec Acteon™, Merignac, France). They were also given a brief questionnaire to ask about factors that may favor dental wear.

The four evaluators were trained both in the use of the Smith and Knight index and in the specific technique required by the intraoral scanner. To carry out the wear assessment, visual inspection using the Smith and Knight index was carried out first to prevent the digital registers from influencing the visual analysis.

### 2.3. Intraoral Scanner Registration Technique

Registration was then carried out using the 3M™ True Definition intraoral scanner (ESPE, Seefeld, Germany).

The scanner used has the following technical characteristics:

- Dimensions of the sensor: length 254 mm, width at the tip 16.2 mm, height 14.4 mm, maximum diameter 24.3 mm, and cable length 2 mm.
- Tip height 14.4 mm, maximum diameter 24.3 mm, and cable length 2 mm. It does not require calibration, which brings convenience to the process.
- The working characteristics of the sensor are: field of view 10 mm × 13 mm, working depth 0 mm to 17 mm, captures 60 images per second, size of touch screen 546.1 mm.

The patient was seated with the back of the dental chair in an upright position and the occlusal plane parallel to the floor to ensure correct registration of the arches to each other. An Optragate™ Ivoclar Vivadent AG (Schaan, Liechtenstein) mouth opener was used to facilitate the scanning of the arches with the intraoral scanner, eliminating soft-tissue interferences. The surfaces to be scanned were covered with titanium oxide powder in the form of Lava Powder spray (3M ESPE, Seefeld, Germany) applied using the Lava Sprayer (3M, Seefeld, Germany) with a 1 mm tip.

Occlusal, incisal, vestibular, lingual, or palatal surfaces were scanned and assigned a wear scale according to the Smith and Knight index criteria (Table 2).

Those volunteers were considered “affected” who presented detailed wear that, according to the Smith and Knight scale, showed values of 1, 2, 3, or 4. The wear of three dental surfaces (vestibular, lingual or palatal, incisal or occlusal) was collected for each tooth. In the case of doubt, when assigning a value according to Smith and Knight dental tissue loss of surface, the lowest value was chosen. In the same way, according to the scanner, those losses of tissue of 200 microns were considered pathological since smaller than this are considered physiological. Values: According to the Smith and Knight test, 1, 2, 3, and 4. According to scanner, 100–200 microns.

At 6 and 12 months, the participants were examined again, following the protocol described above: clinical examination, removal of tartar if necessary, cleaning of possible plaque and/or food debris with a brush mounted on the micromotor, visual registration with the Smith and Knight index, and registration with the intraoral scanner. All results were evaluated in terms of whether there had been a change in the wear quantification scale. It was recorded if volunteers had changes in tooth wear between controls for both assessments by visual inspection and analysis by intraoral scanner.

**Figure 1:**
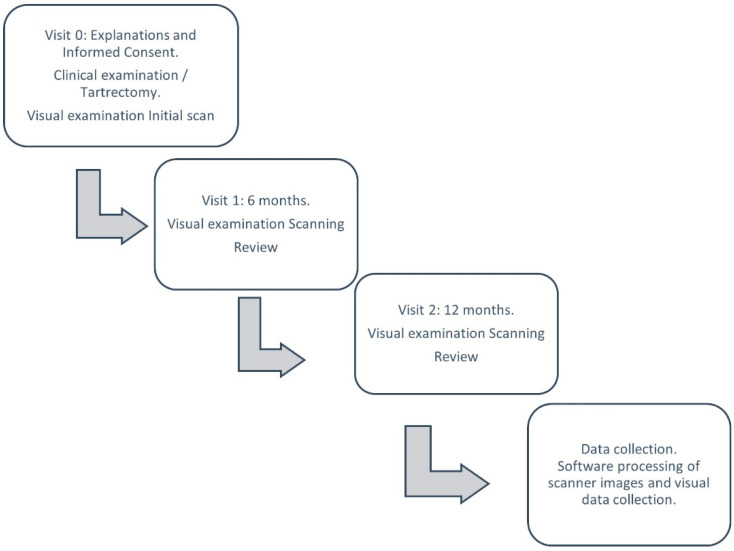
Flow chart of data collection.

The data collection of the study and the scans performed were carried out as shown in Figure 1.

### 2.4. Intraoral Scanner Data Processing

**Figure 2:**
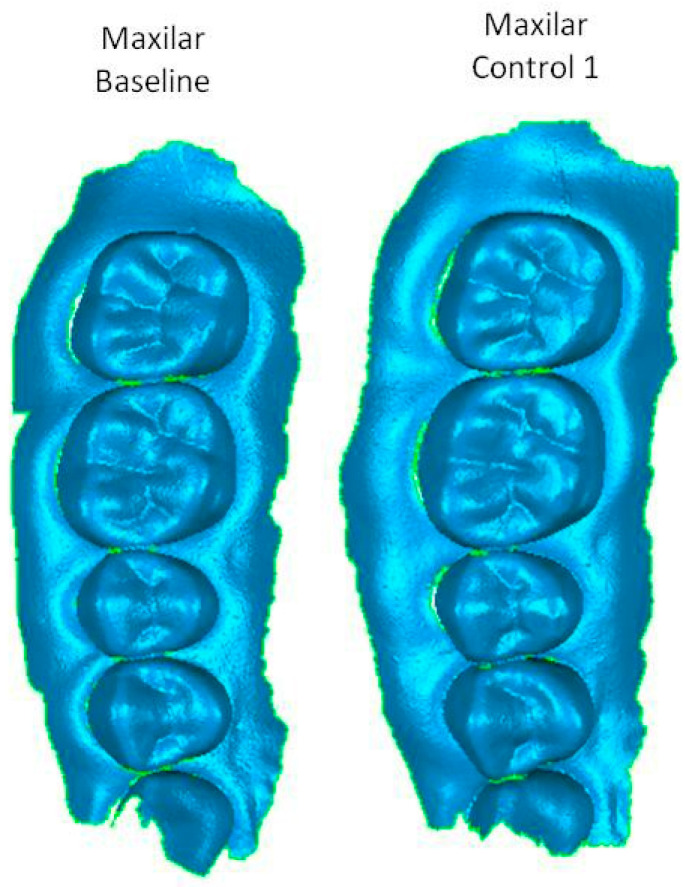
Control images.

All visual log data were stored in an Excel spreadsheet. All scans (*n* = 138) were stored in STL format and processed using Geomatic™ software (3Dsystems, Darmstadt, Germany). The first patient scan was named ‘Baseline’ (*n* = 46), the second scan at 6 months *‘Control 1′* (*n* = 46), and the third scan at 12 months as *‘Control 2′* (*n* = 46) (Figure 2).

**Figure 3:**
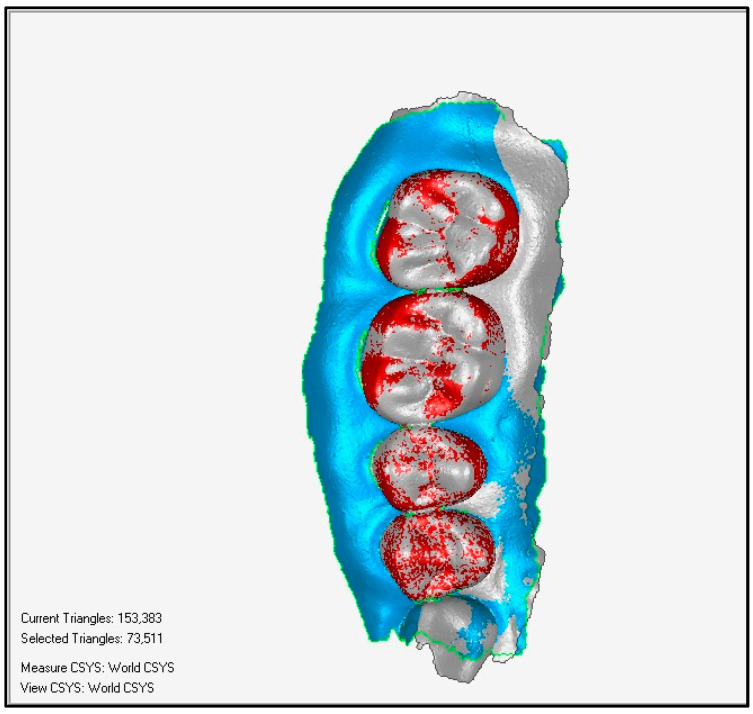
Superimposition of baseline with *Control 1*.

**Figure 4:**
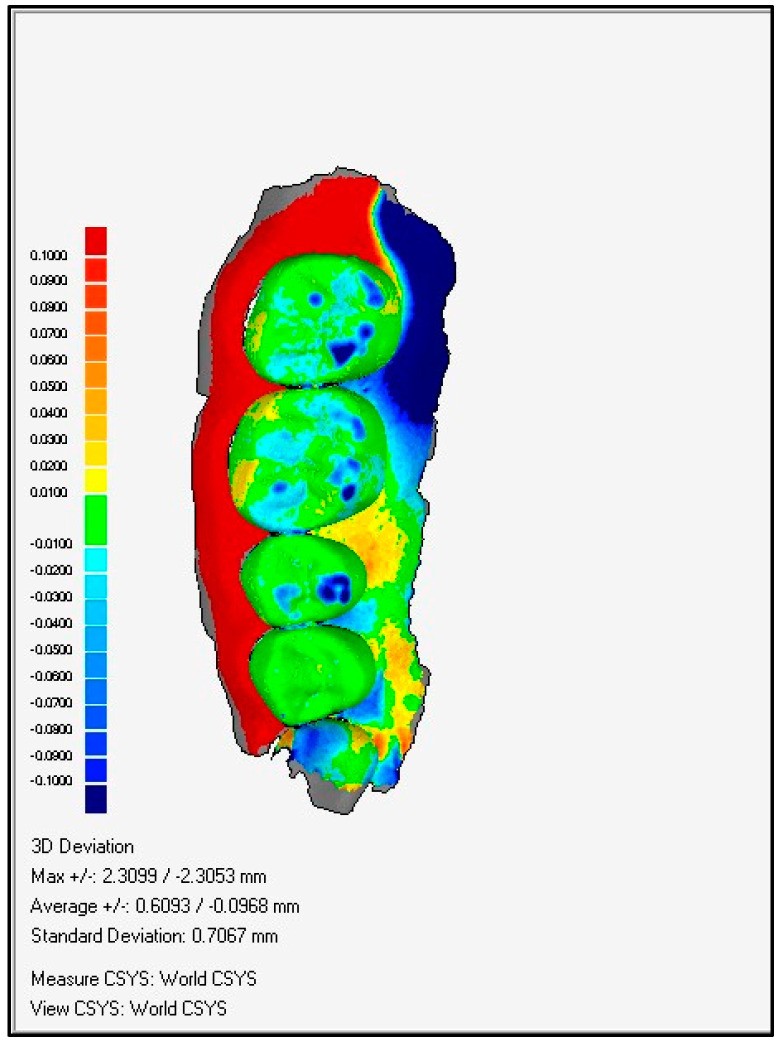
3D comparing.

Through a colorimetric scale of the software, the worn surfaces were evaluated with the following methodology:

1. Overlay and alignment of baseline scan with each successive control scan (Figure 3).
2. Three-dimensional comparison between scans, generating a surface color map showing the differences found, their location on the tooth surface, as well as the intensity of the differences found (Figure 4).
3. A color change scale was used to quantify the dental wear in four areas: antero-superior, postero-superior, antero-inferior, and postero-inferior. This change of scale quantifies variations in dental wear between evaluations (*Baseline*-*Control 1* and *Control 1*-*Control 2)* based on the Smith and Knight index, the reference index for the quantification of dental wear used in the present study.

### 2.5. Satisfaction Survey

Once all the information had been compared and extracted, a survey was carried out among the participants to find out their experience with the True Definition Scanner^®^ and the specific software. It consisted of two items, the first on the usefulness of the scanner as a diagnostic tool and the second on the comfort of the scanning technique. It was scored on a scale of 0 to 5, from ’least’ to ’most satisfied’.

**Figure 5:**
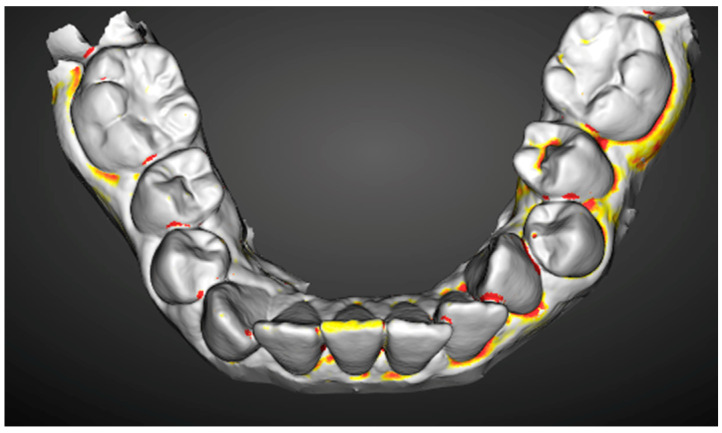
Example of scan presented to the patient.

As an example, Figure 5 shows a complete lower arch, where the patient can observe, thanks to the color graduation of each area, the wear that is being produced.

### 2.6. Statistical Analysis

The study variable, dental wear, was treated as an ordinal qualitative variable, using absolute and relative frequencies to classify the different degrees of wear and the mean ± standard deviation (SD) to determine the degree of general wear. Statistical analyses were performed with the SPSS package (version 21.0. IBM Corp; Armonk, NY, USA).

**Figure 6:**
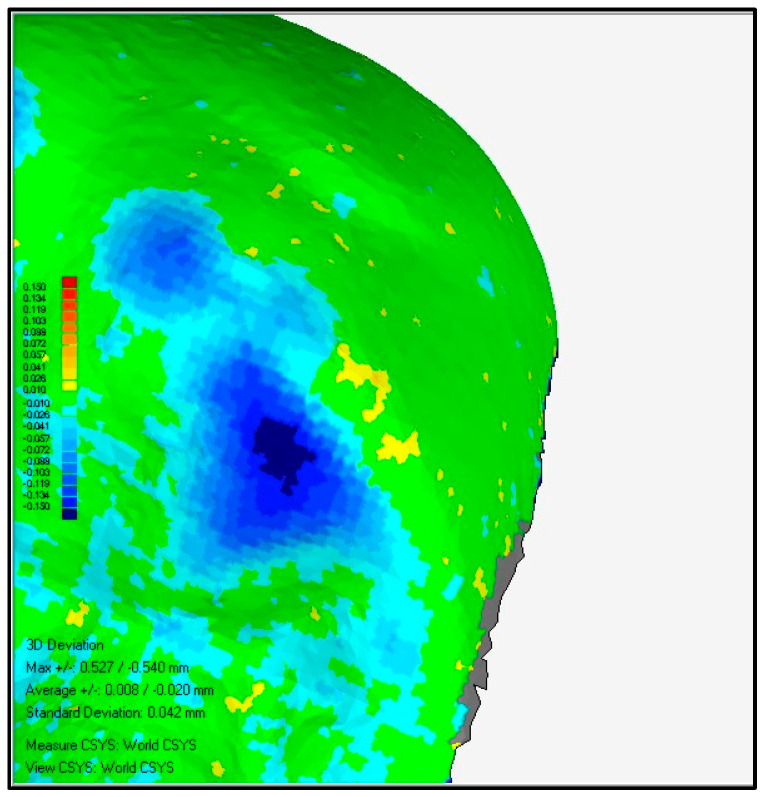
Example of a signal gradient image with clearly defined level lines.

After their correct alignment, they were compared three-dimensionally, generating a colorimetric map showing the differences, their location, and intensity (Figure 5). Teeth with more than 200 microns of dimensional discrepancy were identified. The concept of discrepancy had to meet four conditions:

- That it corresponded to a wear and, therefore, should have a negative value (using the *Baseline* scan as a reference and the control scan as a test).
- It should have a high signal/noise ratio (the value found should be very different from the differences found in the rest of the tooth surface analyzed).
- That it presented a clear signal gradient (clearly defined level lines) (Figure 6).
- That it was maintained or increased in successive control scans.

Sensitivity, specificity, and positive and negative predictive values were assessed to evaluate the efficacy of the scanner. The data collected at each moment of the measurement were used to calculate the sensitivity and specificity. For their preparation, the Smith and Knight visual test was used as a reference for patients and non-diseased patients (considering those classified in stages 1–4 as diseased), and as a diagnostic test was used to validate the 3M^®^ True Definition intraoral scanner (healthy or negative result: 0, and diseased or positive result: 1–4).

## 3. Results

Forty-six volunteers were evaluated for dental wear. There was no loss of patients during the observation period. The short questionnaire conducted prior to the tooth wear assessment showed that 66.7% used some type of dental splint, and 54.35% admitted having bad habits, the most common habit being chewing materials (80%).

### 3.1. Tooth Wear Evaluation

When volunteers were evaluated with the Smith and Knight index, in *Control 1,* the group that presented the greatest wear change was the antero-superior group, while the group with the highest wear in *Control 2* was the postero-superior (Table 3).

The recording of dental wear with the intraoral scanner showed that the dental group that presented the greatest wear in *Control 1* at 6 months was the posterior-superior group (97.56%), while in *Control 2,* the posterior-superior group (100%) and the posterior-inferior group were the dental groups with the highest wear (Table 4).

### 3.2. New Quantification Scale with Intraoral Scanner

Based on the data obtained from intraoral scanner recordings, the authors propose a new way of quantifying tooth wear using the scanner. This classification, which is independent of the one used in Table 3 and Table 4, based on the changes in the scale of the Smith and Knight index, considers that there is pathology and, therefore, a change in scale between controls, if more than 25% of the surfaces are affected. According to this proposed quantification scale, the area most affected by wear in *Control 1* and *Control 2* was the postero-superior, in which wear was 39.0% and 80.5%, respectively. In the rest of the locations, the percentages were 2.2% in antero-inferior, 2.4% in antero-superior, and 17.1% for postero-inferior in the Control 1 period. In the analysis from the beginning to the end of the study, wear in the rest of the locations was 20% for antero-inferior, 19.5% in antero-superior, and 36.1% for postero-inferior (Table 5).

With the obtained results, an accuracy index was performed to measure the percentage of correctly diagnosed patients, treating positives and negatives (true or false) equally. To perform this accuracy index, occlusal surfaces (as they present a less uniform distribution of powder due to tooth anatomy) and lingual/palatal surfaces (because of the tongue handling and presence of saliva) were selected because these conditions may interfere with the scanning. As shown in Table 6, the overall sensitivity and predictive values were 100%, while the specificity and positive predictive values decreased to 84.9% and 71.0%, respectively.

Depending on the surface, as described in Table 7 and Table 8, the predictive value for palatal/lingual and occlusal surfaces were 58.6% and 59.8%, respectively. In addition, regarding the occlusal surfaces, the lowest specificity was recorded with 83.5%.

### 3.3. Satisfaction Survey

**Figure 7:**
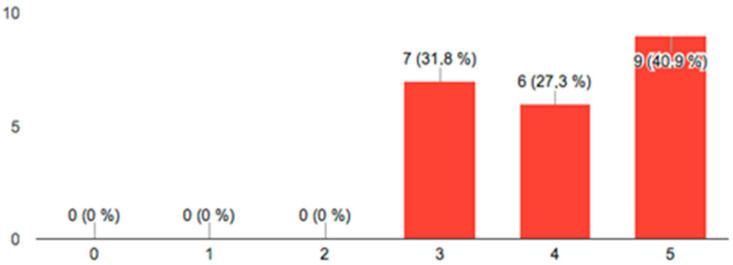
Ratings from 0 to 5 on the usefulness of the scanner as a diagnostic tool.

**Figure 8:**
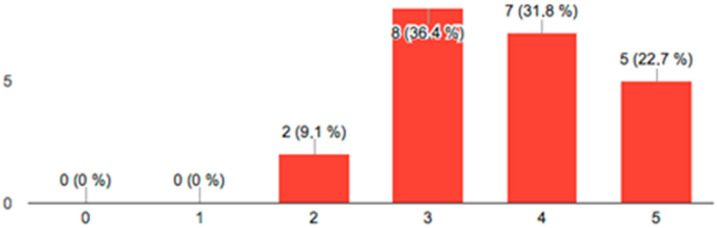
Ratings from 0 to 5 on the comfort of the scanning technique.

Figure 7 shows the results on the usefulness of the scanner as a diagnostic tool, where values higher than three can be seen in all answers, as well as in Figure 8, where the answers on the comfort of the scanning technique are shown.

## 4. Discussion

This study analyzed the sensitivity and specificity of the intraoral scanner as a tool for measuring dental wear. It was observed that the measurement of dental wear with the intraoral scanner presented high percentages of sensitivity and specificity. Therefore, an intraoral scanner could be an alternative diagnostic tool. Tooth wear is an increasingly prevalent pathology in the population [24]; thus, early diagnosis is important to prevent further problems [25]. However, it is complex to reach an early diagnosis due to its multifactorial etiology and the lack of objective detection methods [26]. Milosevic [27] stated that there was no perfect index to satisfy and bring together clinical and research requirements. The implementation of the intraoral scanner in daily clinical practice is reflected in the current scientific literature. Digitization by the intraoral scanner allows direct scanning of the oral cavity [28]. Thus, the present investigation proposes a diagnostic protocol focused on the use of the intraoral scanner as an alternative diagnostic tool for tooth wear detection, as well as other studies that have been published in the literature.

When analyzing tooth wear with the intraoral scanner, high percentages of sensitivity and specificity were observed. Marro et al. [29] evaluated the intraoral scanner to quantify the progression of tooth wear. As in the present study, they observed that the sensitivity levels of the intraoral scanner to detect intraoral wear were higher than the specificity levels. Kumar et al. [15] analyzed the sensitivity of the same intraoral scanner used in the present study for the detection of early tooth wear. They observed that the intraoral scanner could be a promising method for the detection of advanced tooth wear, but that it may have limitations for the detection of early tooth wear. However, Michou et al. [30] observed that the intraoral scanner could detect and monitor early tooth wear.

However, many of the published studies [15,29,30] have been performed in vitro, unlike the present study, which involved volunteers. Thus, in the present study, it was possible to observe the behavior of the intraoral scanner in a real clinical situation. Bastos et al. [23] evaluated the reliability of the intraoral scanner for the assessment of tooth wear in vivo. As in the present investigation, they observed that the intraoral scanner was a reliable tool for the evaluation of tooth wear compared to traditional methods. Furthermore, Esquivel-Upshaw et al. [31] found the intraoral scanner to be an acceptable method for the direct measurement of tooth wear.

With the intraoral scanner, ranges of 200 microns can be achieved, as opposed to classic indexes such as the Smith and Knight visual index, where the scale jump is 1 mm. Within the parameters of the scan and the advantages of the precision that the intraoral scanner can offer, a new quantification scale has been proposed that considers that wear of more than 25% of the affected surfaces between appointments should be considered pathological. In this way, the assessment of tooth wear might be done in a more objective way. When dental wear was evaluated with the intraoral scanner, considering pathological wear, the groups of teeth that presented the highest wear in *Control 2* were the postero-inferior group and the postero-superior group. Meanwhile, when evaluating wear by visual inspection, the group that presented greater wear was the postero-superior group.

Only a few published studies have analyzed the wear of dental groups in adults using the intraoral scanner. Therefore, comparisons were made with studies that evaluated tooth wear in adults using visual indexes. Seligman et al. [32] specified a 91.5% prevalence of bruxing in a university population where patients were evaluated with a scale like the one used in the present study. Jaeggi et al. [33] observed that the occlusal surfaces of mandibular first molars were the most affected, followed by the vestibular surfaces of the maxillary anterior teeth. In contrast to the present study, Marro et al. [34] observed that the distribution of wear was most frequent in the upper incisors, followed by the lateral incisors and canines. However, other studies observed that the prevalence of wear according to location was 52.2% for canines, 41% for incisors, 39.0% for molars, and 17.7% for premolars. Mayhew et al. [35] found 77% attrition in canines due to occlusal guidance. When the patient group explored function, the literature reflects findings of 73.5% in premolars, with respect to 52.83% and 47.17% in the first and second premolars, respectively. This data agrees with the present study since facets were found in 71% of the records in upper teeth and 64% in lower teeth.

Although there is no consensus in the scientific literature on a “gold standard” for the clinical evaluation of wear, the contribution of the indices to diagnosis is undeniable [36]. In the present study, the Smith and Knight index [10] was used as the traditional method, which is the most endorsed by the scientific literature for being quantitative, reproducible, and reliable, as agreed by the European Federation of Conservative Dentistry in 2015 [37]. Furthermore, this index was the first to record that tooth wear is multifactorial, to consider the measurement of tooth wear independently of its etiology, and to distinguish normal from pathological levels. However, one of the limitations might be related to the time involved in applying it. Hence, many other indexes have been developed from this one [10]. Indices such as the Basic Erosive Wear Examination (BEWE) may be easier for the dentist because of its simplicity and comparability [38]. The results of this index show a similar distribution to those of Smith and Knight [39], being an effective test for the most severe cases. However, due to the moderate levels it presents in examiner reliability, it is suggested to interpret the values with caution [40]. In addition, the BEWE index also does not record dentin exposure in detail [41]. For these reasons, in the present study, the BEWE index was discarded.

Literature shows that in modern societies, attrition values originating in young adults are important [42,43]. In the present study, dental wear was investigated in a sample of adults between 20 and 34 years. Nevertheless, other authors extensively studied tooth wear in younger populations. Studies on young adult patients showed contradictory results. Ab Halim et al. [44], using the BEWE method, observed type 2 values when analyzing tooth wear in 16-year-old patients. Similar to the present study, the lower posterior teeth were the most affected. Furthermore, Arnadottir et al. [45], using the Smith and Knight index, found the highest type 2 values at the lower right molar (4.3%) and at the lower left molar (4.2%). However, Larsen et al. [46] chose a pre-university sample and localized the upper antero-incisal as the group most affected by wear. One of the major disadvantages of comparing results between studies is the lack of a standardized methodology. Comparison between studies is difficult due to variability in sample size, indices used [33,47], study designs [47], and examiners [33]. Consequently, when considering the entire dentition, the prevalence of dental attrition presents great variability [34].

The use of the intraoral scanner as a communication tool with the patient is currently considered an item of great importance [48]. In the present study, to evaluate the intraoral scanner as a communication tool, a survey was conducted. The results obtained in the satisfaction surveys were positive for the two items asked. Results showed that there were no scores lower than 3 (on a scale of 0 to 5), suggesting that the scanner appears to be an effective communication tool due to the simplicity with which the colors show the wear and tear produced. The way of perceiving health in contemporary societies makes the patient aware of the oral condition and the treatment needed, being a recurrent problem in daily clinical practice [49]. The use of new technologies in diagnosis allows better patient access to information, an advantage that can be used by the dentist to improve daily clinical practice [50]. On the other hand, in the conventional process of obtaining study models for the diagnosis of wear, many patients report discomfort when taking intraoral impressions, especially those who have difficulty breathing through the nose or those who have an increased gag reflex. Burzynski et al. [51] obtained similar results to those of the present study when comparing the use of two scanner models with conventional impressions in orthodontic patients when only the digital scanning technique was used instead of the conventional one. Siqueira et al. [52] observed that the use of intraoral scanning could improve patient expediency. Nevertheless, Grünheid et al. [53] found that although the time in the entire conventional process was longer than that of scanning, the impression was taken faster; 73.3% of patients preferred the speed of conventional impressions. Furthermore, the use of digital tools promotes sustainability [54] since it eliminates waste generated by the impressions used to obtain the diagnostic models used to evaluate wear.

The present study has some limitations. One limitation is related to the study participants. The sex of the study participants does not represent the entire adult population. Therefore, future studies are needed to analyze the intraoral scanner as a technique in the analysis of dental wear, including a greater heterogeneity of the participants. Furthermore, an additional limitation of the present study is the use of dental splints by 66% of the participants. Considering that the use of dental splints may modify the real dental wear of the patients, the results should be interpreted with caution. Thus, they should be further confirmed with research that analyzes the use of the intraoral scanner for the detection of tooth wear and the influence of splint use on tooth wear.

## 5. Conclusions

With the limitations of the present study, the intraoral scanner showed promising levels of specificity and sensitivity to be used as a diagnostic tool for the measurement of tooth wear. The method for measuring tooth wear proposed in this study is a possible alternative to the visual index; however, it needs to be contrasted with future studies. Volunteer satisfaction with the True Definition Scanner intraoral scanner, both in terms of intraoral recording technique and usefulness as a method of communication with the patient, was positive in all cases.

## Figures and Tables

**Table 1:** Inclusion/exclusion criteria.

Inclusion Criteria	Exclusion Criteria
Over 18 years of age.	Volunteers who do not meet the inclusion criteria.
Students who are not in their last year of undergraduate or graduate studies (potential loss).	Volunteers who, during the study, are diagnosed with such wear that it implies a restorative treatment of both arches with fixed.
Those who are not going to modify their current oral morphology status (orthodontics, surgeries, extractions, fixed, or removable rehabilitations).	Volunteers who are pregnant.
Those who have signed the previous informed consent.	Volunteers who have any kind of academic relationship with the investigators of this study.

**Table 2:** Smith and Knight visual index.

Score	Criteria
0	Vestibular/lingual/incisal or occlusal: no enamel loss or change is observed. Cervical: preserved contour.
1	Vestibular/lingual/incisal or occlusal: loss of surface characteristics. Cervical: minimal loss of contour.
2	Vestibular/lingual or occlusal: loss of enamel (less than 1/3 with dentin exposure). Incisal: loss of enamel with dentin exposure. Cervical: defect less than 1 mm deep.
3	Vestibular/lingual or occlusal: loss of enamel (more than 1/3 with dentin exposure). Incisal: loss of enamel and most of the dentin. Cervical: defect less than 1–2 mm deep.
4	Vestibular/lingual or occlusal: total enamel loss, or exposed pulp or dentin exposure. Incisal: exposed pulp or secondary dentin exposure. Cervical: defect greater than 2 mm, exposed pulp or dentin exposure.

**Table 3:** Smith and Knight (SK) test results.

Parameters	N (%) *Control 1*	N (%) *Control 2*
SK Antero-superior		
No change	41 (90.70)	25 (54.05)
Change of scale	5 (9.30)	21 (45.95)
SK Postero-superior		
No change	43 (93.01)	12 (27.03)
Change of scale	3 (6.99)	34 (72.97)
SK Antero-inferior		
No change	43 (93.02)	25 (54.05)
Change of scale	3 (6.98)	21 (45.95)
SK Postero-inferior		
No change	43 (93.02)	21 (45.95)
Change of scale	3 (6.98)	25 (54.05)

**Table 4:** 3M™ True Definition Scanner results.

Parameters	N (%) *Control 1*	N (%) *Control 2*
TRUE DEFINITION Antero-superior		
No change	18 (39.02)	6 (13.89)
Change of scale	28 (60.98)	40 (86.11)
TRUE DEFINITION Postero-superior		
No change	1 (2.44)	0 (0)
Change of scale	45 (97.56)	46 (100)
TRUE DEFINITION Antero-inferior		
No change	30 (65.85)	9 (19.44)
Change of scale	16 (34.15)	37 (80.56)
TRUE DEFINITION Postero-inferior		
No change	10 (21.95)	0 (0)
Change of scale	36 (78.05)	46 (100)

**Table 5:** New wear scale for intraoral scanner.

Parameters	N (%) *Control 1*	N (%) *Control 2*
Antero-superior		
No change	45 (97.6)	37 (80.5)
Change of scale	1 (2.4)	9 (19.5)
Postero-superior		
No change	28 (61)	9 (19.5)
Change of scale	18 (39)	37 (80.5)
Antero-inferior		
No change	45 (97.8)	37 (80)
Change of scale	1 (2.2)	9 (20)
Postero-inferior		
No change	38 (82.9)	29 (63.9)
Change of scale	8 (17.1)	17 (36.1)

**Table 6:** Overall predictive values.

Parameters	Percentage	CI 95%
SENSITIVITY	100%	99.5–100%
SPECIFICITY	84.9%	83.4–86.4%
PREDICTIVE VALUE +	71.0%	68.3–73.6%
PREDICTIVE VALUE −	100%	99.8–100%
ACCURACY	89.0%	87.8–90.1%

**Table 7:** Predictive values in lingual or palatal faces.

Parameters	Percentage	CI 95%
SENSITIVITY	91.2%	89.1–92.9%
SPECIFICITY	58.6%	51.4–65.4%
PREDICTIVE VALUE +	100%	99.5–100.0%
PREDICTIVE VALUE −	922%	90.3–93.7%
ACCURACY	100%	96.6–100%

**Table 8:** Predictive values in occlusal faces.

Parameters	Percentage	CI 95%
SENSITIVITY	100%	98.7–100%
SPECIFICITY	83.5%	81.3–85.5%
PREDICTIVE VALUE +	59.8%	55.4–64.1%
PREDICTIVE VALUE −	100%	99.6–100.0%
ACCURACY	86.8%	84.9–88.4%

## Data Availability

The data presented in this study are available on request from the corresponding author. The data are not publicly available due to health data protection policies.
